# Uterus Transplantation as Infertility Treatment in Gynecological Cancer Survivors: A Systematic Review

**DOI:** 10.3390/jcm13113172

**Published:** 2024-05-28

**Authors:** Ermioni Tsarna, Anna Eleftheriades, Alkis Matsas, Olga Triantafyllidou, Panagiotis Christopoulos

**Affiliations:** 1 Second Department of Obstetrics and Gynecology, Faculty of Medicine, “Aretaieion” Hospital, National and Kapodistrian University of Athens, 11528 Athens, Greece; ermioni.tsarna@gmail.com (E.T.); amatsas@med.uoa.gr (A.M.); triantafyllidouolga@gmail.com (O.T.); 2 Postgraduate Programme “Maternal Fetal Medicine”, Medical School, National and Kapodistrian University of Athens, 11527 Athens, Greece; annielefth-28@hotmail.com

**Keywords:** gynecological cancer, cervical cancer, endometrial cancer, ovarian cancer, uterus transplantation, absolute uterine factor infertility, AUFI, cancer survivors

## Abstract

**Background**: The aim of this systematic review is to summarize the evidence regarding the acceptance of uterine transplantation as infertility treatment among gynecological cancer survivors, surgical and pregnancy outcomes post-transplantation for gynecological cancer survivors, as well as relevant adverse events. **Methods**: PubMed and Embase were searched for records published since 2000, and extensive reference screening was performed. **Results**: Out of 1901 unique records identified, 7 are included in this review; 4 examined the proportion of gynecological cancer survivors among applicants for uterine transplantation, 2 examined rejection rates, pregnancy rates, and outcomes after uterine transplantation among gynecological cancer survivors, and 2 reported the frequency of relevant adverse events. Among the applicants, 60/701 (8.6%) were gynecological cancer survivors, only 1 transplanted patient was a cervical cancer survivor and achieved two live births after eight embryo transfers, and 2/27 (7.4%) of uterus transplantation recipients were diagnosed with CIN post-transplantation. **Conclusions**: Uterus transplantation can be regarded as an infertility treatment for absolute uterine factor infertility (AUFI), although only one gynecological cancer survivor has received a uterus transplantation. The efficacy, safety, and ethical considerations for gynecological cancer survivors need to be addressed for uterine transplantation to become an infertility treatment option for AUFI among gynecological cancer survivors.

## 1. Introduction

Gynecological malignancies affect, among others, women of reproductive age, posing a threat to their fertility and future maternity options. Ovarian, uterine, and cervical cancer accounted conjointly for 1,405,661 new cancer cases globally in 2022, corresponding to 7% of all new cancer cases [1]. With regard to cervical cancer in particular, which represents 8% of all new cancer cases, 25% of newly diagnosed women are below 40 years old [2,3]. Fertility-sparing therapeutic options do exist nowadays, but several limitations hinder their widespread application. Regarding uterine cancer, total hysterectomy remains the recommended standard surgical treatment, whereas in cases of cervical cancer, radical hysterectomy is recommended for patients with cervical cancer larger than 2 cm [4,5]. Therefore, women diagnosed with gynecological cancer during their reproductive years often face absolute uterine factor infertility (AUFI), as a result of surgical treatment for cancer.

Ensuring the highest quality of life for cancer survivors, including onco-fertility, has been a very active field of research during the latest years. Recent guidelines describe fertility-preserving options for gynecological cancer patients treated in onco-fertility clinics. With regard to cervical cancer that is commonly diagnosed during reproductive years, fertility-sparing surgical treatment, via conisation or trachelectomy, is considered a safe approach for patients up to stage 1B1 [6]. Even in 1B2 patients, neoadjuvant chemotherapy may be applied, followed by a fertility-sparing surgery [6]. Among ovarian cancer patients, those with non-epithelial tumors, low-grade stage IA, and selected IC1 stages may undergo unilateral oophorectomy, while the cryopreservation of gametes is advised for fertility preservation [7]. Lastly, younger grade 1 endometrial cancer patients can be managed conservatively with progestins and postpone hysterectomy and salpingo-oophorectomy until after giving birth [8]. However, this is still considered as a non-standard treatment, and patients must be informed accordingly [8]. Despite the aforementioned advances in onco-fertility, disease staging prerequisites and the lack of access to onco-fertility clinics contribute to AUFI among reproductive-aged gynecological cancer survivors.

AUFI describes the complete absence of a uterus in a female patient leading to an inability to achieve gestational motherhood. AUFI is estimated to affect 1 in 500 women of reproductive age, which translates to approximately 200,000 women in Europe [9]. Causes of AUFI may be grouped into congenital and acquired categories [10]. Congenital causes apply to the patients born without a uterus, the majority of whom are diagnosed with Mayer–Rokitansky–Küster–Hauser (MRKH) syndrome [10]. Acquired AUFI is the result of hysterectomy, which may be performed for benign conditions (e.g., leiomyomas), in cases of severe post-partum hemorrhage, or due to malignancy, as is the case for uterine cancer and, often, cervical cancer [10]. Uterine transplantation represents a novel infertility treatment and is the only one that can offer the opportunity for gestational motherhood to AUFI patients, gynecological cancer survivors among them, who, until recently, could achieve motherhood only via adoption or surrogacy.

The aim of this systematic review is to summarize the available research data and evidence with regard to the acceptance of uterine transplantation as an infertility treatment among gynecological cancer survivors, as well as the surgical and pregnancy outcomes and relevant adverse events after uterus transplantations among gynecological cancer survivors.

## 2. Materials and Methods

In this systematic review, we considered as eligible for inclusion all peer-reviewed research papers and posters presented in scientific conferences that examined uterine transplantation in adult survivors of gynecological cancer. In particular, we aimed to include records that reported the proportion of cancer survivors among applicants for uterine transplantation, records that examined rejection rates, pregnancy rates, and pregnancy outcomes after uterine transplantation among gynecological cancer survivors, and records that reported frequency rates of adverse events after successful uterine transplantation, particularly relevant for cancer survivors. Even though adult patients with other causes of absolute uterine factor infertility were regarded as the ideal comparator group, studies without a comparator group were also included in this systematic review. With regard to the publication type, all records reporting results from randomized controlled trials, cohort studies, cross-sectional studies, case series, and case reports were considered as eligible for this systematic review. In contrast, records were excluded if they were literature reviews, systematic reviews and/or meta-analyses, editorials, letters to the editor if they did not summarize a study, and records for which the full text was not available. Finally, records reporting results from animal studies and records published in a language other than English, German, or Greek were also excluded from this systematic review.

In order to identify records eligible for our systematic review, we searched PubMed and Embase for records published after 1 January 2000, since in 2000, the first uterus transplantation attempt in humans took place in Saudi Arabia, up to September 2023 [11]. The initial search algorithm was developed in PubMed and utilized both searching keywords in records’ titles and abstracts and searching using relevant Mesh terms. In particular, keywords and Mesh terms that describe cancer and uterus transplantation were included with appropriate Boolean operators, and a filter was applied to safely remove all animal studies. The search algorithm in PubMed is presented in detail in Table 1. The afore-presented search algorithm was translated for Embase, with the use of the Polyglot Search tool from Systematic Review Accelerator, and is presented in Appendix A—Table A1 [12].

All records retrieved from PubMed and Embase after applying our search algorithm were imported in Rayyan, where deduplication was performed [13]. Subsequently, two independent reviewers assessed titles and abstracts and excluded only the records that clearly did not meet our inclusion and exclusion criteria. In cases of discrepancy, the record’s full text was assessed for eligibility. The full text from potentially eligible records was assessed by two independent reviewers; discrepancies were solved via discussion and consent, and a senior reviewer’s opinion was taken into consideration in cases of disagreement. To identify other potentially relevant reports that were not retrieved from our search algorithm in PubMed and Embase, two independent reviewers manually searched, following a similar process as the one described above, the reference list from all reports that were assessed in full-text form, even if they were finally excluded. In addition, we searched for reports of long-term results for uterine transplantation patients with a history of gynecological cancer that participated in already-identified trials.

To summarize and synthesize the reviewed research data and evidence, research papers and posters were grouped based on the examined outcomes as follows: records that reported the proportion of gynecological cancer survivors among applicants for uterine transplantation, records that examined rejection rates, pregnancy rates, and outcomes, and, finally, records that examined adverse events after surgically successful uterine transplantation. Two independent reviewers retrieved and tabulated relevant data from all included research papers and posters, namely, we recorded the type of publication (peer-reviewed paper or poster), the year of publication, the type of study (clinical trial, case series, case report, etc.), the country where the study was performed, the sample size, details regarding the gynecological cancer and previously applied therapeutic strategies for cancer survivors, the study’s examined outcomes, and the study’s findings. In addition, inclusion and exclusion criteria were recorded in cases of data arising from clinical trials. The aforementioned tabulated data were compared to identify potential discrepancies, in which case a third senior reviewer independently tabulated the same data, and agreement was achieved via group discussion among the authors and consent. This systematic review contains a qualitative but not a quantitative summary of the findings because of the expected small number of relevant records per outcome and the relatively small sample size. With regard to a risk-of-bias assessment, a specific tool was not used for this purpose due to the expected heterogeneity in the studies’ design that would not allow for the use of one tool for all records and, thus, a comparison of the risk of bias across studies. However, a group discussion took place among the authors with regard to potential biases among the reviewed studies, and identified sources of bias are presented in our results and discussion. The reporting of this systematic review follows the PRISMA guidelines [14].

## 3. Results

**Figure 1:**
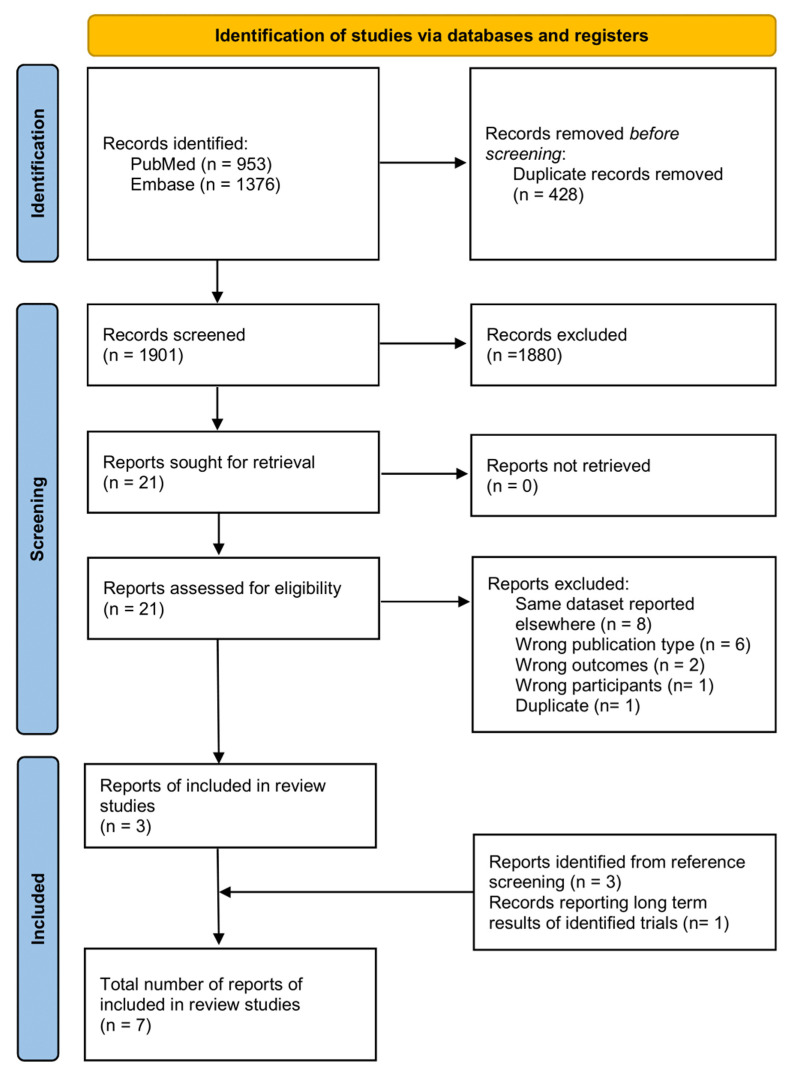
Flow diagram showing the number of titles and abstracts identified and screened and full-text research papers assessed for eligibility and included in the qualitative synthesis.

Our search algorithm identified 953 records from PubMed and 1376 records from Embase, of which 428 were duplicates, leading to 1901 unique records. After screening titles and abstracts, 1880 records were excluded, as they clearly did not meet our inclusion and exclusion criteria, while 21 reports were sought for retrieval, and the full text was accessed for all of them. After screening their full text for eligibility, 18 were excluded, 8 because they reported results from the same datasets as other included studies, 6 due to the wrong publication type (5 reviews and 1 editorial), 2 due to them being irrelevant for our review outcomes, 1 due to a lack of gynecological cancer survivor participants, and 1 because it was a duplicate that was not detected while screening titles and abstracts. In addition to the three included studies, three reports were identified from reference screening and one report with long-term results of an already identified trial, leading to a total of seven reports of studies that are included in this systematic review (Figure 1). Of these seven included reports, one was a poster from a scientific conference and the remaining six were papers published in peer-reviewed scientific journals (Table 2) [15,16,17,18,19,20,21]. Four of those reports examined the proportion of gynecological cancer survivors among applicants for uterine transplantation, two reports presented results from the same trial, one examining rejection rates post-transplantation and the other examining pregnancy rates and live birth rates after uterine transplantation among gynecological cancer survivors, and, finally, two studies reported the frequency of adverse events after uterine transplantation that are particularly relevant for cancer survivors (Table 2) [15,16,17,18,19,20,21].

With regard to the acceptance of uterine transplantation as an infertility treatment for AUFI among gynecological cancer survivors, the results from four peer-reviewed research papers are summarized here [15,16,17,20]. In all four studies, 701 women applied for uterine transplantation, of whom 60 were gynecological cancer survivors, corresponding to 8.6% [15,16,17,20]. However, there was great heterogeneity in the results from individual studies, as gynecologic cancer survivors represented 7.2% of applicants in the French study, 6.1% and 16.3% of applicants in the studies from the USA, and 0% in the Turkish study [15,16,17,20]. On one hand, this heterogeneity might represent a real difference in the acceptance of uterine transplantation among gynecological cancer survivors across different cultures and healthcare systems. Notably, the authors of the study from the USA were themselves surprised with their results, as the majority of applicants suffered from acquired AUFI secondary to previous hysterectomy (64%) rather than congenital AUFI (32%), as they expected [16]. On the other hand, this heterogeneity might represent differential bias affecting individual studies’ results. Since uterine transplantation is a novel infertility treatment for AUFI that has gained great media attention, it is expected that applicants are influenced by media presentation and/or recruitment methods that were utilized in each study. Therefore, if congenital AUFI has gained more attention in local media and/or recruitment strategies, women suffering from acquired AUFI might have been implicitly discouraged from applying for uterine transplantation.

With regard to research data for the efficacy of uterine transplantation when applied in gynecological cancer survivors, only one such patient from Sweden has been identified, and relevant results were reported in two peer-reviewed research papers [18,19]. Although it is impossible to draw safe conclusions regarding the efficacy of a method from data arising from only 1 participant among 70 uterus transplantations performed worldwide to date (corresponding to 1.4% of uterus transplantation recipients), we will summarize the clinical course of this patient, which has been successful [22]. At 25 years of age, which was seven years before the uterus transplantation, she had undergone a radical hysterectomy and pelvic lymph node dissection because of a 3 cm-sized cervical cancer (stage 1B1); the lymph nodes were proven to be negative in a pathology report [23]. Post-hysterectomy, the patient was consistently negative for HPV, based on vaginal cytology, and had no evidence of disease [23]. She received a uterus from a living donor—specifically, her mother [18]. Post-transplantation, she received mycophenolate mofetil and tacrolimus for six months and monotherapy with tacrolimus thereafter; she experienced only one mild rejection episode during the second month post-transplantation [18]. The patient had regular menstrual bleeding starting within two months post-transplantation, and the Doppler examination of the anastomosed uterine arteries was within the normal range, taking into consideration the menstrual cycle phase throughout the study period [18]. She achieved her first pregnancy after six embryo transfers (success rate 17%), leading to a planned live birth at 35 weeks of gestation, and a second pregnancy after two embryo transfers (success rate 50%), leading to a planned live birth at 37 weeks of gestation, after which a hysterectomy took place [19].

With regard to adverse events after surgically successful uterine transplantation, two reports are included in this systematic review; one is a poster presented in a scientific conference and the other is a paper published in a peer-reviewed journal [18,21]. These two studies included 27 patients in total from Sweden and the USA and emphasized adverse events linked to the immunosuppressive drugs administered post-transplantation. Gastrointestinal complaints, nephrotoxicity, hepatotoxicity, and infections (both bacterial and viral) were reported; these occurred in 25% of patients in the USA trial [21]. As particularly relevant for gynecological cancer survivors, we regarded the two reported cases of cervical intraepithelial neoplasia (CIN) as a result of HPV infection (2/27, corresponding to 7.4%) [18,21]. The follow-up period ranged from 12 months post-transplantation for the study from Sweden to 24–60 months for the study from the USA [18,21]. Notably, the patient in the Swedish trial had to undergo loop excision due to CIN II, whereas the applied surveillance and/or therapeutic strategy was not reported for the patient with CIN I participating in the trial from the USA [18,21].

## 4. Discussion

In this systematic review of the literature, uterine transplantation was examined as infertility treatment for AUFI among gynecological cancer survivors. Even though gynecologic cancer survivors represent 8.6% of women applying for uterine transplantation, indicating high acceptance of the method, they represent only 1.4% of uterine transplantation recipients. With regard to adverse events, CIN as a result of HPV infection in medically immunosuppressed patients represents a worth-mentioning risk, especially for cervical cancer survivors.

As professor Mats Brännström, a pioneer of the uterus transplantation field, has recognized, the concept of uterus transplantation was first suggested by a cervical cancer patient in 1998 when she faced permanent AUFI due to radical hysterectomy [24]. Notably, in 1998, the first larynx transplantation took place, which introduced quality-of-life-enhancing transplantations along with the life-saving transplantations that had long been performed [25]. Nonetheless, to date, only one gynecological cancer survivor with AUFI has received a uterus transplantation, despite the fact that 1,405,661 new cervical, uterine, and ovarian cancer cases were diagnosed globally in 2022 and research among uterus transplantation applicants has indicated that these patients are interested in uterus transplantation as infertility treatment for their AUFI [1].

The great discrepancy between the proportion of gynecological cancer survivors among uterus transplantation applicants and recipients needs to be addressed. Several restrictions in the inclusion and exclusion criteria of clinical trials might explain this discrepancy, to an extent. For example, in Dallas uterus transplantation trial, intact ovarian function and a BMI ≤ 30 were among the inclusion criteria, and a similar BMI exclusion criterion has been applied in the clinical trial in Germany [26,27]. These criteria, however, would have been unachievable for the majority of endometrial cancer survivors with AUFI, who would have undergone bilateral oophorectomy as part of their treatment and are likely to suffer from obesity, a well-known risk factor for endometrial cancer [26]. Intact ovarian function would also be unachievable for the majority of ovarian cancer survivors, including patients who have been managed in onco-fertility clinics according to ESMO guidelines [7]. Furthermore, the age limit for recipients has been consistently set below 40 years old, which is less than the current age limit for IVF in many countries [22]. However, gynecological cancer survivors also need to be at least five years free of disease for safety reasons. That leads to constricting uterus transplantation to gynecological cancer survivors that have been diagnosed well before 35 years of age. This does not imply that these inclusion and exclusion criteria are unreasonable but rather underlines how these contribute to limiting current uterus transplantation recipients to patients with other causes of AUFI rather than prior gynecological cancer.

Uterus transplantation is nowadays still mainly performed in research settings. To reach the approved number of participants, researchers often have to choose among numerous applicants, as indicated by publications regarding the applicants’ characteristics [15,16,17,20]. Participants are often selected among a pool of applicants that all comply with inclusion and exclusion criteria based on a lower surgical risk for adverse events and a higher probability of uterus transplantation success, even though this is not explicitly stated. The risk for surgical complications, long-term failure of transplantation, and/or failure of subsequent IVF can be considerably higher among gynecological cancer survivors for several reasons. To begin with, post-hysterectomy adhesions might have developed, which increases the risk for intraoperative complications. This holds true for all post-hysterectomy patients and is not specific to gynecological cancer survivors, but it might, at least partially, explain why the majority of patients who have received uterus transplantation suffer from congenital rather than acquired AUFI. In addition, many gynecological cancer survivors have also undergone pelvic irradiation, in which case side effects on pelvic vessels might threaten the anastomoses success and lead to severe morbidity [28]. Both radiotherapy and chemotherapy, which a gynecological cancer survivor might have received, are also linked to a decreased ovarian reserve, which would severely impact the patient’s probability to achieve genetic motherhood [15]. Notably, a similar argument can be raised for all cases of prior hysterectomy, even for benign conditions, since the consequent decrease in ovarian circulation is thought to negatively affect the ovarian reserve, even when compared to patients with congenital AUFI [17,29]. Lastly, in all cases of severely affected ovarian function or ovarian absence due to prior bilateral oophorectomy, the only benefit of uterine transplantation over surrogacy or adoption would be achieving gestational motherhood, a situation raising ethical issues in terms of the risk–benefit ratio.

In order for uterus transplantation to become an infertility treatment option for gynecological cancer survivors, the issue of a decreased ovarian reserve has to be effectively addressed. It is, however, impossible to avoid oophorectomy, hysterectomy, radiotherapy, and chemotherapy for all gynecological cancer patients, as that would directly endanger achieving optimal or at least acceptable therapeutic outcomes for these patients [15,17,29]. Nonetheless, assisted reproduction techniques, such as oocyte retrieval and oocyte or embryo cryopreservation applied before the aforementioned cancer treatments, provide a realistic strategy to this end. Even for patients for whom uterus transplantation might be judged to be technically impossible after cancer treatments, the cryopreserved oocytes or embryos can be used to achieve genetic motherhood via surrogacy.

With regard to the safety of uterus transplantation in gynecological cancer survivors, several considerations that are particularly relevant for this group of AUFI patients are thought to contribute to their exclusion to date. In particular, the risk for cancer relapse or newly diagnosed cancer as a result of the immunosuppressive regimen administered post-transplantation is of interest. Due to the relatively low number of uterus transplantations performed to date, relevant data do not exist and the aforementioned considerations are drawn from extrapolating data form other solid organ transplantation recipients. In kidney transplantation recipients, the risk for HPV-related cancers, namely, cervical, vulvar, and anal cancer, has been estimated to be considerably higher compared to that of the non-transplanted population, while the overall cancer risk is reported to be 4.8% during the first five years and steadily increases thereafter [30,31]. More importantly, all-cause and cancer-specific mortality, as well as getting diagnosed with a new malignancy, are greater among the subgroup of solid organ recipients with a previously diagnosed cancer compared to those without [32]. However, a younger age at kidney transplantation has been shown to be a protective factor against cancer diagnosis, which increases steeply after the first decade of treatment with immunosuppressive drugs [33]. Taking into consideration that uterus transplantation recipients are younger compared to other solid organ transplantation recipients and that immunosuppressive treatment is administered for a limited period of time until one or two pregnancies with live births are achieved, the aforementioned cancer-related risks are expected to be less pronounced than in lifelong organ transplantations. Notably, attempts have been made to further decrease the time interval to embryo transfer after uterus transplantation by eliminating mycophenolate mofetil from the immunosuppressive regimen and generating embryos before the uterus transplantation takes place [34].

With regard to ethical considerations, compared to other forms of solid organ transplantations, uterus transplantations have a short history, and ethical frameworks are based largely on existing guidelines for other solid organ transplantations. However, uterus transplantation differs from other forms of solid organ transplantations in several important respects. For example, uterus transplantations are temporary, as the transplanted uterus is removed after the recipient has completed their family planning, and unlike other solid organ transplantations, they are not life-saving, life-extending, nor directly life-enhancing; thus, one can argue that the transplantation provides no direct health benefits to the recipient [35]. There is currently no consensus regarding who should be able to participate in uterus transplantation research trials, leading to a great variability in inclusion and exclusion criteria internationally. For example, the Swedish criteria explicitly require recipients to live in a stable couple relationship. Conversely, based on the UK criteria, recipients who already have children are excluded, even in cases of children who have been adopted or born via surrogacy arrangements, while the Swedish criteria appear to focus only on biological parenthood. These inconsistencies point towards the ethical complexity of developing inclusion and exclusion criteria for uterus transplantation clinical trials [36,37].

There are also some specific risks associated with uterus transplantations, particularly in light of their reproductive purposes, that are not seen in other solid organ transplantations—for example, risks related to the immunosuppression affecting fertility and/or pregnancy outcomes [38]. Post-transplantation, the recipients will have to undergo a long process and a series of interventions, since pregnancy following uterus transplantation relies on assisted reproductive technology because the fallopian tubes are not transplanted. Prior to uterus transplantation, recipients create embryos through in vitro fertilization (IVF) that can be transferred after uterus transplantation. Other interventions include intense, continuous immunotherapy, major and prolonged transplantation surgery, and gestation with ongoing careful monitoring for graft rejection. Potential drawbacks are also psychological risks, such as guilt for graft failure, pregnancy loss, or prematurity, and possible disappointment with the reality of a physical pregnancy without innervation, cesarean delivery, and, finally, hysterectomy [37]. In the case of patients who received uterus transplantation due to previous gynecological cancer, these difficulties would be even greater in the context of a previous abdominal operation and its potential complications, such as adhesions, the existence of scar tissue, etc.

Uterus transplantation also carries risks to the resulting children, who would be subjected to immunosuppressive therapy while in utero [39]. The exposure of the developing fetus to immunosuppressive medications throughout gestation may also have unforeseen health consequences, such as transgenerational epigenetic effects, the consequences of which may not be known for decades. In many documented cases, there was also the need for greater bolus doses and different immunosuppressive regimens mid-gestation to avoid the risk of uterine rejection. A study summarizing the initial outcomes of the first 5 years of uterus transplantation in the US also concluded that 24% of such pregnancies developed gestational hypertension, potentially due to vascular insufficiency [38]. In cases of gynecological cancer survivors, the participation in such trials should perhaps take into consideration the life expectancy of the mother as well as the risk for the relapse of her initial malignancy. Since pregnancy following a uterus transplantation relies on assisted reproductive technology, an additional ethical consideration is raised regarding preimplantation genetic diagnosis for evaluating the genetic profile of the fetus, in cases of gynecological malignancies with a strong genetic background.

To our knowledge, this is the first study that systematically reviewed research data and evidence regarding uterine transplantation as an infertility treatment among gynecological cancer survivors. Among the strengths of our systematic review is the broad search algorithm that was applied as well as the extensive search for additional records. Even so, this systematic review also has several limitations. The study’s protocol was not registered in Prospero; nonetheless, the only change during the systematic review process was searching for reports of long term results for uterine transplantation patients with a history of gynecological cancer that participated in already-identified trials. With regard to our literature search strategy, a language restriction was applied, which led to the exclusion of a potentially relevant report in French regarding the proportion of gynecological cancer survivors among uterine transplantation applicants, which was identified from manual reference screening. In addition, grey literature was searched only via Embase; however, we believe that this should suffice, with respect to publication bias, since the results from novel therapeutic interventions, such as a uterine transplantation, are not likely to be underreported, even in cases of negative results. The small number of included studies and the corresponding small sample size pose an impregnable obstacle to drawing conclusions regarding the safety and efficacy of uterine transplantation among gynecological cancer survivors as an infertility treatment and do not allow for a formal assessment of publication bias (e.g., via a funnel plot). Additionally, in our systematic review, we focused on AUFI among gynecological cancer survivors and the issue of relative uterine factor infertility due to a decreased uterine blood flow, decreased uterine size, and atrophic endometrium after pelvic or whole-body irradiation was not discussed because of the lack of relevant data [40]. Lastly, owing to the heterogeneity in the study design and examined outcomes, a formal risk-of-bias assessment was not feasible. Nonetheless, sources of bias were extensively discussed among the authors and have been presented.

## 5. Conclusions

Uterus transplantation can be regarded as an infertility treatment for AUFI that has been proven to be successful in research settings. However, to date, it is mainly patients with congenital AUFI who have benefitted from this therapeutic option, while only one gynecological cancer survivor has received a uterus transplantation leading to two live births. Research among applicants for uterus transplantation indicates a considerably more widespread interest among gynecological cancer survivors. Nonetheless, the efficacy, safety, and ethical considerations for this group of patients need to be addressed for uterine transplantation to become an infertility treatment option for AUFI among gynecological cancer survivors.

## Figures and Tables

**Table 1:** The search algorithm in PubMed.

(“cancer”[tiab] OR “neoplas*”[tiab] OR “malignan*”[tiab] OR “proliferat*”[tiab] OR “Neoplasms”[Mesh] OR “Cancer Survivors”[Mesh] OR “Uterine Neoplasms”[Mesh] OR “Uterine Cervical Neoplasms”[Mesh] OR “Ovarian Neoplasms”[Mesh])
**AND**
(“uterine transplant*”[tiab] OR “uterus transplant*”[tiab] OR ((“Uterus”[Mesh] OR “uterus” [tiab] OR “Womb”[tiab] OR “Uterine”[tiab]) AND (“transplantation”[MeSH] OR “Transplants”[Mesh] OR “transplant recipient*”[MeSH] OR “tissue donor*”[MeSH] OR “transplant*”[tiab] OR “graft*”[tiab])))
**NOT**
(animals [mh] NOT humans [mh])

An asterisk (*) in PubMed is the truncation symbol and represents any group of characters, including no character; it is used in search algorithms in order to search for all terms that begin with that basic word root.

**Table 2:** Summary of findings of the reviewed studies.

	Original Study	Country	Type of Study	Sample Size	Number of Gynecological Cancer Survivors	Gynecological Cancer	Cancer Therapies	Examined Outcomes	Findings
Peer-reviewed papers	Huet et al., 2016 [15]	France	Observational Study	39	10	Ovarian cancer (*n* = 4), cervical cancer (*n* = 3), bladder sarcoma (*n* = 1), and choriocarcinoma (*n* = 1)	Hysterectomy before 8.37 years (range 3–23); bilateral adnexectomy in five patients	Proportion of gynecological cancer survivors among uterus transplantation applicants	7.20%
	Arian et al., 2017 [16]	USA	Observational Study	239	39	Not specified	Not specified	Proportion of gynecological cancer survivors among uterus transplantation applicants	16.30%
	Akar et al., 2015 [17]	Turkey	Observational Study	144	0	Not applicable	Not applicable	Proportion of gynecological cancer survivors among uterus transplantation applicants	0%
	Johannesson et al., 2018 [20]	USA	Observational Study	179	11	Not specified	Hysterectomy	Proportion of gynecological cancer survivors among uterus transplantation applicants	6.10%
	Johannesson et al., 2015 [18]	Sweden	Clinical Trial	7	1	Cervical Cancer (stage 1B1)	Radical hysterectomy and pelvic lymph node dissection	Post-transplantation menstruation, uterine artery blood flow, rejection episodes, and relevant adverse effects of immunosuppression post-transplantation	Menstruation within two months post-transplantation, normal Doppler studies of uterine arteries, and no rejection episodeCIN II in one out of seven patients (14.3%)
	Brännström et al., 2022 [19]	Sweden	Clinical Trial	7	1	Cervical Cancer (stage 1B1)	Radical hysterectomy and pelvic lymph node dissection	Pregnancy rates and live birth rates per embryo transfer post-transplantation	First pregnancy leading to live birth after six embryo transfers (rate 17%) Second pregnancy leading to live birth after two embryo transfers (rate 50%)
Poster	Johannesson et al., 2022 [21]	USA	Clinical Trial	20	0	Not applicable	Not applicable	Relevant adverse effects of immunosuppression post-transplantation	CIN I in 1/20 patients (5%)

## Data Availability

Not applicable.

## Appendix A

**Table A1:** The search algorithm in Embase.

(cancer:ti,ab OR neoplas*:ti,ab OR malignan*:ti,ab OR proliferat*:ti,ab OR Neoplasms/exp OR ‘Cancer Survivors’/exp OR ‘Uterine Neoplasms’/exp OR ‘Uterine Cervical Neoplasms’/exp OR ‘Ovarian Neoplasms’/exp)
**AND**
(‘uterine transplant*’:ti,ab OR ‘uterus transplant*’:ti,ab OR ((Uterus/exp OR uterus:ti,ab OR Womb:ti,ab OR Uterine:ti,ab) AND (transplantation/exp OR Transplants/exp OR ‘transplant recipient*’/exp OR ‘tissue donor*’/exp OR transplant*:ti,ab OR graft*:ti,ab)))
**NOT**
(animals/exp NOT humans/exp)

An asterisk (*) in PubMed is the truncation symbol and represents any group of characters, including no character; it is used in search algorithms in order to search for all terms that begin with that basic word root.
